# Why Different Results Follow the Same Line of Treatment in Similar Pathological Conditions in the Oral Cavity

**Published:** 1892-08

**Authors:** J. D. Patterson

**Affiliations:** Kansas City, Mo.


ARTICLE V.

### WHY DIFFERENT RESULTS FOLLOW THE SAME LINE OF TREATMENT IN SIMILAR PATHOLOGICAL CONDITIONS IN THE ORAL CAVITY.

BY J. D. PATTERSON, D. D. S., KANSAS CITY, MO.

**Read at the annual meeting of the Missouri State Dental Association, at Clinton, Mo., July, 1892.**

You must agree with me when I say that in our reading of dental literature and especially in discussion before society meetings we are confronted with the fact that the similar treatment of similar pathological conditions are said to bring about such complex, diverse and hetrogenous results. We are often constrained to wonder which of the two, the operator or the patient, presents such curious idiosyncrasies. As knowledge increases and as every year should bring to the dental practitioner a more perfect science in combatting disease, it is therefore of prime interest and importance why these apparently unexplainable results should follow.

In looking carefully over the field it must be admitted that a not inconsiderable factor in bringing about these curious results must be found in the fallibility of human testimony. It *may* be on account of the fact that the operator may not have been accustomed to carefully weigh his words, or be possessed with the idea that he must antagonize his *confrere* and assent dogmatically what he only surmises.

In a given pathological condition one operator details a certain treatment with a certain result. His neighbor may in a similar case with similar treatment have a different result. Now in endeavoring to harmonize these opposite statements we almost invariably look for different physical conditions, and, if we do not find them, we are apt to think the matter is not explainable. If, however, we were to study the two operators who have made these two opposing statements possibly the difficulty would be easily solved. The method of one of them although stated to be the same as the other, is found really to have no parallel at all. The education of one of them has been defective—his manipulative ability and scientific knowledge has no parallel in the other and more highly trained hand and mind—and therefore his evidence cannot be said to be reliable. He will tell you that he did thus and so,—and the result was not the natural sequence to be expected—when the fact has been that he did not do so at all. He did not mean to speak falsely, but at the same time he *did* speak falsely. He said that he had perfectly removed all cause of irritation, and in medication used the remedies scientifically. His better educated confrere would find neither of his statements true, but at the same time must criticise his education and not his honesty. So I apprehend, as at first I intimated, that a not inconsiderable reason for the diverse results from the same treatment is found in this fallibility of testimony which is the result of a difference in the acquirements of the operator. This feature of the question, however, can perhaps only be considered as subjective. The chief study of the question suggested by the title of this article may be found in the study of the tissues upon which the operator labors, rather than in the study of the operator himself.

In the surgery of the mouth we find that in inflammatory conditions certain prescribed and standard treatment seems at times to have little or no effect, while again, the relief is instantaneous, and careful study results in the knowledge that tissue conditions potently control, limit and assist the curative efforts of the operator.

It is found that every human body presents differences which must affect our efforts in combating disease. The condition in the life of the tissue as viewed from a standpoint of perfection where every departure from normal would, under treatment, have only one result, presents to the student such varied manifestations that a continual research is necessary in order that the operator shall suit his treatment to the character of tissue upon which he works. Every man, woman, or child presents to an outward view certain peculiarities of appearance—certain distinctive movements in mind and body, that we can well believe that similar differences must be found in the tissues which compose their bodies. We find in constitutional temperaments and their sub-divisions a marked difference in tissue character, and is it then still wonderful that similar treatments in similar troubles give diverse results? If you will insist upon treating a highly nervous, encephalic patient in the same manner and with the same strength of dose as you would a rugged, bilious or lymphatic one you must expect dissimilar and unsatisfactory results. Every human tissue, hard or soft, adipose or cellular, has come into existence perhaps under different conditions in its formative stage, and some difference in the cell perfection is found. It may have been surrounded by predisposing influence, such as weak nutrition, low vitality, and the result is that the cell elements are lacking in perfect material and ever afterward are unable with great vital force to throw off disease attacks.

This condition in our patients can be diagnosed by appearances, general and minute, showing in general the lack of tonicity and vigor, and in the tissue an inability to throw off local irritant attacks, by judging of the constitutional temperament and following out in treatment that knowledge which the study brings.

Again, in all pathological conditions the susceptibility of tissue to cure is widely influenced by *acquired*, hereditary or constitutional conditions—cachexia—in which a depraved bodily habit exists *e.g.* the scrofulous, cancerous, the syphilitic. This condition demands close observation, scrutiny and skill, if we would serve our patients well who are suffering from specific blood taint. We will not enter into the discussion as to whether the scrofulous, the leprous and the cancerous all belong to the syphilitic, but only to the consideration of the class which so far as our specialty is concerned may be considered as one class.

Those who have been careful observers must have noticed how difficult and disheartening is the attempt to allay pericemental irritation or cure inflammation of the mucous membrane, when there is the syphilitic or scorbutic condition. The abscess which we are treating goes very slowly to a cure, and drainage must be left open longer in these cases. There must be extreme care in each detail of the operation so that irritation is reduced to the minimum. Where tissue is healthy a considerable amount of morbid matter may not cause irritation, but where a cachexia exists the least amount will cause inflammation. In these cases also, after all has been done that skill and patience can devise and the permanent root filling inserted, it will often be found that continued or periodical chronic soreness will remain for months or years. In the cavity of the antrum where dental irritation has had its seat and the patient is syphilitic I have had two cases where I have finally despaired of a permanent cure, the trouble recurring when the bodily condition fell into one of the characteristic low periods which is so familiar to students of that disease.

The result we account for by finding that in these tissue conditions the ability to ward off disease is a weak tone; the vital safeguards are wanting, and when the tissue is invaded the lymphatics and absorbents are so crippled by their imperfect structure that effete and irritant matter is very slowly carried away or absorbed. The practitioner then should be perfectly conversant with the physical signs which denote a cachexia, so that added care and skill shall be given such cases. In medication in these cases the general rule must be that more time and a greater amount of germicides, disinfectants and stimulants must be used, and this always in conjunction with remedies internally administered and strict hygenic supervision. In all the departments of dental practice I think that in these cases perhaps greater acumen is necessary than in any other work which we are called upon to do.

Another important reason why anticipated results do not appear in treatment is the degree in which the tissue may be *locally* perverted. In neglected abscess for example, (and I make no apology for again instancing abscess; for it is the most usual pathological condition we are brought in contact with) when it has run its course and has been long neglected we find the neighborhood surrounding the apical portion of the tooth impregnated with morbid matter and inflammatory effusions. The healthy tissue is gone and in its place we find the perfection and continuity of tissue broken and infiltrated with depraved cell elements, which poison and irritate. There are irregular patches of repair tissue where a struggle has been made by nature for a cure, but without avail until surgical science has assisted. The history of these cases is generally misleading and we often fail in their treatment because we do not diagnose a long standing diseased condition. The immediate root filler who treats each case of pulpless teeth in one way will especially be brought to see how *painfully* different are the results which follow his treatment.

When this class of cases is found in cachetic patients the complication is still harder to treat and the utmost skill, knowledge and patience is necessary. After a cure is effected and new tissue in patches takes the place of the original, it is a tissue which is always under suspicion and gives way quickly under renewed irritation. In all repair tissue the cell elements are perverted, the vascular and nervous supply is irregular, constricted, and its power to throw off irritation is small. This tissue will, under debilitating influences of health and climate, give evidence of its condition in low grade pain without local irritation. A familiar example is seen in a tooth which has been restored after long disease and loss of tissue and around which there is much repair tissue and process. These are cases which require the most scrupulous care and judgment.

Another class of cases still which give almost surely a negative result, are those where disease has long surrounded the apex of the root until perverted cells have attacked the surface of the root, eating deep irregular burrows, leaving sharp spicula-like edges, spiking the new tissue forming until inflammation results. The extreme difficulty with these cases is that we have no means by which we can judge whether such a condition is present or not and therefore we grope blindly until extraction shows the trouble. The surgical operation of amputating and smoothing the ragged end is the only operation that is available.

In summing up upon the subject we see therefore that in pathological conditions of the oral cavity, we must consider a great variety of factors to determine why certain results come, or do not come under treatment.

1st. The intelligence of the operator.

2d. Hereditary constitutional conditions.

3d. Acquired conditions of tissue.

4th. Cachexia.

5th. Local tissue conditions acquired by local disease.

The study of the subject is one of practical interest, and when fully comprehended will enable us to reach success when hitherto we have oftentimes been confronted with failure.—*Western Dental Journal.*

